# First-Time Diagnosis and Ultrasound Features of Extranodal Lymphoma in Children

**DOI:** 10.1155/2022/4229112

**Published:** 2022-05-12

**Authors:** Na Li, Min Li, Qian Zhang, Jingli Wei, Bailing Liu

**Affiliations:** Department of Ultrasound, Xi'an Children Hospital, Xi'an 710003, Shaanxi Province, China

## Abstract

**Objective:**

To assess the diagnostic value of ultrasound for extranodal lymphoma in children.

**Methods:**

In this retrospective study, the first diagnostic clinical manifestation, ultrasound sonograms, and puncture pathology of 40 cases of pediatric lymphoma treated in Xi'an Children's Hospital from August 2018 to March 2020 were analyzed.

**Results:**

The first diagnostic clinical manifestation varied from jaundice to gastrointestinal, motor, neurological, respiratory, circulatory skin, and soft tissue multisystem disorders. The intranodal ultrasound results showed abnormally enlarged lymph nodes with extranodal involvement of the liver, pancreas, spleen, kidney, ovaries, and mediastinum as hypoechoic masses with multiple plasma cavity effusions, and color Doppler flow imaging (CDFI) results showed abundant blood flow signals in the lesions. The pathological diagnosis by ultrasound-guided puncture was Hodgkin lymphoma, non-Hodgkin lymphoma, and multiple subtypes.

**Conclusion:**

Routine ultrasound can provide an imaging basis for early identification and differential diagnosis of lymphoma, and ultrasound-guided puncture biopsy is simple, minimally invasive, and histopathological-based.

## 1. Introdution

Lymphoma is a malignant tumor of the lymphatic system with an incidence of up to 40% in extranodal nodes [1]. The initial diagnostic manifestations of extranodal lymphoma in children are complicated and non-specific, and the limited verbal expressions of children result in an increased chance of clinical misdiagnosis and underdiagnosis, which necessitates effective imaging diagnosis [2, 3]. Ultrasonography is simple and nonradioactive and has been widely used in the routine examination of pediatric diseases. In recent years, with the continuous improvement of color Doppler ultrasound technology, its application has been extended to the clinical diagnosis of extranodal lymphoma. Therefore, this study used color Doppler ultrasound to diagnose children with extranodal lymphoma. The limitation of this study is the absence of diagnostic efficacy testing, which will be further investigated in future studies. Here, the clinical and ultrasonographic data of 40 cases of children with extranodal lymphoma were retrospectively analyzed, aiming to improve the early diagnosis and differential diagnosis of children with extranodal lymphoma by ultrasound.

## 2. Materials and Methods

### 2.1. Clinical Data

In this retrospective study, 40 cases of pediatric lymphoma treated in Xi'an Children's Hospital were assessed for eligibility and recruited between August 2018 and March 2020. The study was reviewed and approved by the ethics committee of the Xi'an Children's Hospital, and all children and their families gave an informed consent, with an ethics approval number of 2017-11-12.

### 2.2. Apparatus and Methods

#### 2.2.1. Ultrasound Examination

The color Doppler diagnostic ultrasound instrument (EPIQ 7) was used for examinations, with a convex array probe frequency of 5-8 MHz and a linear array probe frequency of 9-18 MHz. In conjunction with the clinical manifestations of the children, a comprehensive scan was performed in multiple examination positions of the children. The focus, frequency, two-dimensionality, and depth of the instrument were adjusted appropriately for different parts of the examination until the ultrasound image was of the best quality and the required standard dynamic, flow, and cross-sectional images were retained. The corresponding sonograms were saved in the ultrasound image management system to provide the necessary information for subsequent analysis.

## 3. Results

### 3.1. **Baseline Data and** Primary Sites

40 children with lymphoma were recruited, including 31 males and 9 females, aged from 5 months 1 day to 14 years 10 months, with a median age of 5.8 years. All diagnosis of the children was confirmed by puncture biopsy or surgical pathology. Among the 40 cases of extranodal lymphoma, there were 8 cases of Hodgkin's lymphoma (HL) (5 cases of nodular sclerosis, 2 cases of mixed cell type, and 1 case of other types) and 32 cases of non-Hodgkin's lymphoma (NHL), including 12 cases of Burkitt's lymphoma (BL), 8 cases of mesenchymal large cell lymphoma (LBCL), 8 cases of lymphoblastic lymphoma (LBL), and 4 cases of other lymphomas. The primary sites of the 40 cases of extranodal lymphoma were the intestinal wall, liver, gastric wall, mediastinum, pancreas, kidney, ovary, and skin and subcutaneous tissue (Table 1).

### 3.2. First Diagnostic Clinical Manifestations of 40 Cases of Extranodal Lymphoma

The first diagnostic clinical manifestations of 40 cases of extranodal lymphoma include 26 cases of gastrointestinal symptoms (including 16 cases of abdominal mass, 4 cases of acute abdomen, 4 cases of chronic abdominal pain, and 2 cases of bloody stool), 4 cases of jaundice (including 1 case of vomiting blood), 6 cases of motor or neurological symptoms (including 5 cases of lower limb mobility disorders and 1 case of foot pain), 6 cases of respiratory symptoms (including 3 cases of respiratory distress), and 2 cases of skin and soft group lesions.

### 3.3. Ultrasound Imaging

**Figure 1:**
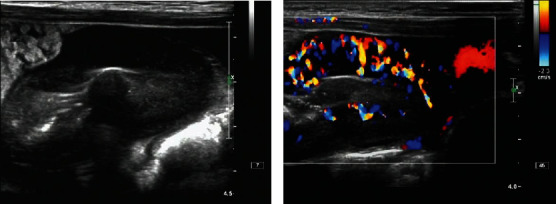
(Extranodal) ileal lymphoma. (a) Significant thickening of the intestinal wall at the end of the ileum, with uneven thickness, reduced internal echogenicity, and reduced intestinal lumen. (b) CDFI shows a rich and disturbed blood flow pattern in the intestinal wall.

Of the 40 cases of extranodal lymphoma, 22 cases were found in the intestinal canal. Ultrasound showed eccentric thickening of the intestinal wall in 10 cases with centripetal, whole wall uniform or heterogeneous thickening, loss of hierarchical structure, more homogeneous hypoechoic, and reduction of the intestinal lumen, and the color Doppler flow imaging (CDFI) demonstrated abundant blood flow signal in the intestinal wall. Also, ultrasound showed 12 cases of localized masses, which were manifested as abdominal intussusception sonograms, with hypoechoic or excessively hypoechoic masses with irregular morphology visible in the wall of the intestinal canal in the sleeve, and the CDFI showed scanty to moderate blood flow signals (Figure 1).

The ultrasound of the carcinoma at the primary sites of the pancreas (2 cases) and kidney (2 cases) showed hypoechoic or excessively hypoechoic masses in the parenchymal organs, which exhibited rich blood flow signals on CDFI.

**Figure 2:**
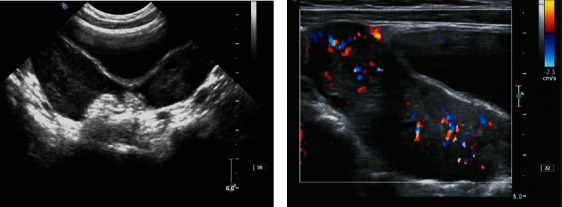
(Extranodal) ovarian lymphoma. (a) Bilateral ovarian enlargement as a hypoechoic mass with irregular morphology and localized multiple follicular echogenicities. (b) CDFI shows a rich blood flow signal in the mass.

The ultrasound of the carcinoma at the primary sites of ovaries (2 cases) showed bilateral ovarian enlargement with hypoechoic and irregular morphology and localized multiple follicular echogenicities, and CDFI showed a rich blood flow signal in the ovaries (Figure 2).

The ultrasound of the carcinoma at the primary sites of the mediastinum (3 cases) showed irregular hypoechoic with close relation to the thymus and relatively homogeneous and excessively hypoechoic, without calcification or liquefaction, and CDFI showed a punctate blood flow signal.

**Figure 3:**
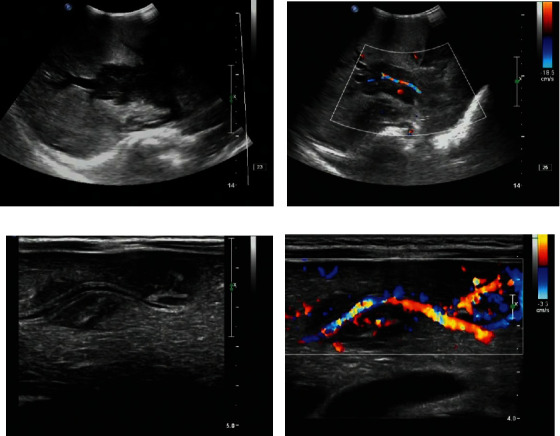
(Extranodal) hepatic lymphoma. (a) Hypoechoic mass in the portal region of the liver with irregular morphology, encircling the portal sector veins, and bile ducts in a cuffing sign. (b) CDFI shows extrusion and thinning of the main trunk of the portal vein. (c) Multiple hypoechoic intrahepatic masses encircling and traveling along the Glison sheath, showing a cuffing sign. (d) CDFI shows the signal of portal vein branching blood flow.

The ultrasound of the carcinoma at the primary sites of the liver (4 cases) showed multiple hypoechoic masses with unclear borders and irregular morphology in the liver, involving the intrahepatic Glison sheath in a typical cuffing sign, and CDFI demonstrated a rich blood flow signal. (Figure 3).

The ultrasound of the carcinoma at the primary sites of the gastric wall (4 cases) showed a heterogeneous thickening of the gastric wall with loss of hierarchical structure and more homogeneous hypoechogenicity, and CDFI demonstrated abundant blood flow signal in the corresponding area of the gastric wall.

There were two cases of primary sites at the skin and soft tissue lesions, which showed local ulcer formation.

## 4. Discussion

In recent years, the incidence of lymphoma in children shows an upward trend due to various factors such as environmental changes, and there is a significant gender difference, with a male-to-female ratio of about 3 to 5 : 1 [4, 5]. In the present study, the male-to-female ratio was approximately 3.4 : 1 (31 cases:9 cases), which is basically consistent with the previous literature. Lymphoma may develop in the body where there are lymphatic vessels, lymphatic organs, and lymphatic tissues [6, 7]. The initial diagnostic manifestations of intranodal lymphoma are mostly chronic, progressive, and painless lymph node enlargement, while those of extranodal lymphoma mainly consist of diverse and nonspecific symptoms of each involved organ and system. Thus, early diagnosis is essential to improve the patient's prognosis [8].

In the present study, 5 cases had recurrent intermittent enlargement and reduction of cervical lymph nodes with irregular fever, which failed to be promptly treated due to parents' negligence. Two cases involved the stomach wall and intestinal wall, respectively, with recurrent bloody stools and anemia at the initial diagnosis and were admitted to the gastroenterology department for clinical diagnosis of peptic ulcer or polyps. Three cases involved the ileocecal intestine and tract secondary to recurrent intussusception and were admitted to the Department of General Surgery for the acute surgical abdomen. One case involved the Glison sheath of the liver, with jaundice and vomiting blood at the first-time diagnosis, and was admitted to the Department of Infection for obstructive jaundice and portal hypertension. Three cases involved the mediastinum and pleura, with cough, breathlessness, and progressive dyspnea at the first diagnosis and were admitted to the Department of Respiratory Medicine or the Department of Cardiology for clinical diagnosis of pneumonia and massive fluid accumulation in one, two, or three chambers. Two cases involved the soft tissues of the maxillofacial region, were manifested as localized masses at the first diagnosis, and were admitted to the Department of Stomatology for mumps and inflammation, respectively. One case involved temporal orbital soft tissue and localized bone destruction and was admitted to the Department of Orthopedics for clinical diagnosis of Langerhans histiocytosis. Two cases were admitted to the Department of Dermatology and the Department of Orthopedics for recurrent ulcerative skin nodules, respectively.

In the present study, the detection rate of lymphoma by conventional ultrasound was 100%. Typical intranodal lymphoma ultrasound showed abnormally enlarged lymph nodes, single or multiple, either large or small, with clear borders, full morphology, longitudinal and transverse diameters <1, extremely low internal echogenicity, excessively hypoechoic, mostly without liquefaction and calcification, and with deviation or disappearance of lymphatic portals. CDFI showed an internal nonlymphoid portal blood supply with abundant blood flow signal. This requires differentiation from necrotizing lymphadenitis, EBV-infected lymphadenitis, and mononucleosis [9]. Extranodal lymphoma involving the mediastinum was characterized by a parenchymal echogenic mass in the mediastinum or thymus with well-defined borders, hypoechoic and heterogeneous internal echogenicity, and some were manifested as a one-, two-, or three-chamber effusion, with poor permeability of the fluid area and a large number of punctate echogenicity. In the case of extranodal lymphatic involvement in the abdominopelvic organs, the ultrasound showed a mass echogenicity of the involved organs, which could be the stomach wall, intestinal wall, and mesentery [10], pancreas, spleen, kidneys, ovaries, and testes [11], with clear or indistinct borders and uneven internal hypoechogenicity. The involvement of the intestinal canal may be associated with intussusception [12], and the involvement of the liver parenchyma or Glison sheath may present as a cuffing sign. The involvement of extranodal lymphoma in the soft tissues of the maxillofacial region was characterized by parenchymal echogenic masses with localized bone destruction, and the involvement of subcutaneous tissue in the skin was manifested by swelling [13]. Therefore, extranodal lymphoma warrants differentiation from other malignancies in children to ensure better treatment efficiency.

The World Health Organization (WHO) divides lymphomas into two main types, HL and NHL. The HL accounts for about 15% of pediatric lymphomas and NHL for about 80%-85%. Each type is also subdivided into different subtypes, of which BL accounts for approximately 40%, LBL accounts for approximately 30%, and large cell lymphoma occupies approximately 25-30% [14]. Histopathological diagnosis is the “gold standard” for the diagnosis of pediatric lymphoma, and histomorphological classification and immunohistochemical phenotyping are available for accurate clinical diagnosis and treatment guidance. For children with lymphoma detected by conventional ultrasound, ultrasound-guided puncture biopsy is rapid, safe, and minimally invasive, which can replace surgical biopsy to obtain histopathological samples [15]. Herein, the success rate of puncture biopsy was 100%, and 8 cases of HL and 56 cases of NHL were pathologically diagnosed, accounting for 12.5% and 87.5% of the cases in the present study, respectively, including 24 cases of BL, 15 cases of LB, and 12 cases of LBCL in NHL, accounting for 42.9%, 26.8%, and 21.4%, respectively, which is basically in accordance with the ratio reported in the literature.

The previous research results showed that the diagnostic accuracy of ultrasound for extra-lymph node lymphoma lesions was 96.08% using puncture biopsy and surgical pathological histological findings as the gold standard, which was significantly higher than that of 78.43% for computed tomography with a statistically significant difference, suggesting a higher diagnostic value of ultrasound in extra-lymph node lymphoma lesions versus computed tomography [16]. However, the diagnostic accuracy of ultrasound and computed tomography was similar in primary gastric lymphoma, intestinal lymphoma, thyroid lymphoma, adrenal lymphoma, hepatic lymphoma, breast lymphoma, and lymphoma. The ultrasound presentation of various types of extra-lymph node lymphoma lesions was more distinct for identification. The diagnosis of patients with extranodal lymphoma lesions using conventional ultrasound technology is of low specificity, especially for extranodal lesions of cavernous organs, such as the primary colon, which have low diagnostic accuracy, whereas the diagnostic accuracy is superior for lymphoma in superficial organ sites, such as the breast. Therefore, for extranodal lymphoma lesions, the puncture biopsy under ultrasound guidance is advisable to ensure clinical diagnostic accuracy and to provide a theoretical basis for the development of reasonable treatment protocols for patients with extranodal lymphoma lesions.

## 5. Conclusion

Routine ultrasound can obtain an imaging basis for early identification and differential diagnosis of lymphoma, and ultrasound-guided puncture biopsy is simple, minimally invasive, and histopathological-based, which contributes to enhancing the precise diagnosis and prognostic assessment of clinicians.

## Figures and Tables

**Table 1:** General data.

Index	Number
Year	
<6 years	21
6-12 years	12
>12 years	7
Hodgkin's lymphoma (HL)	8
Nodular sclerosis	5
Mixed cell type	2
Other	1
Non-Hodgkin's lymphoma (NHL)	32
Burkitt's lymphoma (BL)	12
Mesenchymal large cell lymphoma (LBCL)	8
Lymphoblastic lymphoma (LBL)	8
Other lymphomas	4
Primary sites	
Intestinal wall	22
Liver	4
Gastric wall	3
Mediastinum	3
Pancreas	2
Kidney	2
Ovary	2
Skin and subcutaneous tissue	2

## Data Availability

The datasets used during the present study are available from the corresponding author upon reasonable request.
